# Incidence and Correlates of Maternal Near Miss in Southeast Iran

**DOI:** 10.1155/2015/914713

**Published:** 2015-02-15

**Authors:** Tayebeh Naderi, Shohreh Foroodnia, Samaneh Omidi, Faezeh Samadani, Nouzar Nakhaee

**Affiliations:** ^1^Research Center for Health Services Management, Institute of Futures Studies in Health, Kerman University of Medical Sciences, Kerman 76175-113, Iran; ^2^Research Center for Social Determinants of Health, Institute of Futures Studies in Health, Kerman University of Medical Sciences, Kerman 76175-113, Iran; ^3^Neuroscience Research Center, Institute of Neuropharmacology, Kerman University of Medical Sciences, Kerman 76175-113, Iran

## Abstract

This prospective study aimed to estimate the incidence and associated factors of severe maternal morbidity in southeast Iran. During a 9-month period in 2013, all women referring to eight hospitals for termination of pregnancy as well as women admitted during 42 days after the termination of pregnancy were enrolled into the study. Maternal near miss conditions were defined based on Say et al.'s recommendations. Five hundred and one cases of maternal near miss and 19,908 live births occurred in the study period, yielding a maternal near miss ratio of 25.2 per 1000 live births. This rate was 7.5 and 105 per 1000 in private and tertiary care settings, respectively. The rate of maternal death in near miss cases was 0.40% with a case:fatality ratio of 250 : 1. The most prevalent causes of near miss were severe preeclampsia (27.3%), ectopic pregnancy (18.4%), and abruptio placentae (16.2%). Higher age, higher education, and being primiparous were associated with a higher risk of near miss. Considering the high rate of maternal near miss in referral hospitals, maternal near miss surveillance system should be set up in these hospitals to identify cases of severe maternal morbidity as soon as possible.

## 1. Introduction

There are approximately 287,000 preventable maternal deaths annually, of which 99% occur in developing countries [1]. As a sentinel event, maternal death is also a prime indicator in evaluating the quality of a nation's health care delivery systems [2, 3]. Mothers are pivotal to the social, economic, and cultural development of a community [2]; maintaining their health elevates the physical, psychological, and social well-being of their children and families and, by extension, society as a whole [4]. For this reason, improving maternal health has been proposed by the World Health Organization (WHO) as one of their eight Millennium Development Goals (MDG) [1].

Traditionally, maternal death evaluation has been viewed as key to maternal death prevention [5]. However, in countries with few maternal deaths, this approach fails to provide comprehensive information, leaving policy makers to react based on current rather than past statistics. To facilitate the development of precautionary measures and safer environments that minimize maternal deaths, it is essential that near miss data are recorded and analyzed [6]. The WHO defines an individual having experienced severe acute maternal morbidity (SAMM) (i.e., near miss) as “a woman who nearly died but survived a complication that occurred during pregnancy, childbirth or within 42 days of termination of pregnancy” [7].

In fact, maternal near miss includes those cases in which a woman nearly died but survived during pregnancy or during 42 days after the delivery [8]. Using near miss data in maternal death prevention planning has several advantages. First, because the number of near miss cases exceeds maternal death cases, near miss is a better predicate for preventive planning. Second, because the mother survives a near miss, she can provide valuable details on what she experienced. Lastly, because near miss is one step removed from death, obtaining any information about the event could prove useful in preventing maternal death [8–10].

The prevalence of maternal near miss varies among different countries based on health care quality and availability. Nevertheless, in a systematic review using disease-specific criteria, near miss rates have been reported to be between 0.6% and 14.98% [11].

From a global perspective, Iran has been notably successful in reducing maternal mortality. Between 1990 and 2008, Iran has managed to decrease its maternal mortality rate by 80% [1], which, at present, translates to about 25 deaths in 100,000 [12]. The availability of emergency obstetrical care, improvements in women's education [13], and the expansion of family planning services have all contributed to the decrease in maternal mortality [14]. In Iran, a national maternal mortality surveillance system examines maternal death cases by reviewing files and interviewing key parties to determine the cause of death [15]. Although some studies have been carried out in Iran and the Middle East regarding the causes of maternal death and risk factors of pregnancy [12–17], the authors know of no published study on severe maternal morbidities and near miss occurrences from Iran or other Middle Eastern countries. The present study aimed to establish a profile of severe maternal morbidities in Iran and their relationship with other underlying factors.

## 2. Method

This prospective study encompassed eight hospitals with maternity facilities located in the two large cities of Kerman and Jiroft in southeast Iran. The study was performed in 2013 for a period of nine months. The study protocol was approved by the Ethical Committee of Kerman University of Medical Sciences (E.C./90/518). After explaining the study's nature and aims, oral consent was obtained from the participants who all were ensured that their information would remain confidential. First, a list of maternal near miss conditions was prepared based on Say et al.'s recommendations for prospective surveillance of maternal near miss cases [8]. This list included four major groups of haemorrhagic disorders, hypertensive disorders, severe management indicators, and other systemic disorders. Moreover, a category including other conditions was also considered so as not to miss any other life-threatening illness [8]. All women admitted during the nine-month study period for delivery or completion of pregnancy as well as women admitted within 42 days after the termination of pregnancy were enrolled into the study. After the participating hospitals' maternity, labour, general ICU, emergency, and admission departments were fully coordinated, our case survey was implemented. A check list was completed for cases with potentially life-threatening conditions by a team consisting of a midwife and gynaecologist. The check list was composed of two parts. The first part included demographic and clinical data such as age, educational level, place of residency, gravidity, parity, type of delivery, and gestational age. The second part comprised the list of potentially life-threatening conditions. Next, a woman admitted to the delivery room at the same hospital was randomly selected from the list of patients as the control, and the first part of the check list was completed for her. In all stages, an experienced expert supervised completion of the check lists. According to the WHO definition maternal near miss ratio was defined as “the number of maternal near-miss cases per 1000 live births” [18].

Chi-square and independent *t*-tests were used to compare the qualitative and quantitative variables between near miss and control groups. A stepwise logistic regression model was used to determine the relationship between underlying variables and near miss ratio. The logistic regression model's goodness of fit was evaluated by the Hosmer-Lemeshow test.

## 3. Results

During the study period, there were 501 cases of near miss in 19,908 live births (a near miss ratio of 25.2 per 1000 live births). The highest near miss ratio (104.8 in 1000) was observed in the referral (educational) hospital (Table 1). The mean age of near miss cases was 28.3 ± 6.1 years versus the control group's 26.0 ± 5.8 years (*P* < 0.001). University degrees were seen more among near miss women (Table 2). In the near miss group, 208 women (41.5%) were primiparous, whereas, in the control group, the number was 225 (45.2%; *P* = 0.243). The frequency of abortion in the near miss and control groups was 18.6% and 1.6%, respectively (*P* < 0.001). The frequency of caesarean section in the near miss and control groups was 24.7% and 54.2%, respectively (*P* < 0.001).

In our study group, there were two cases of maternal death. One was a 19-year-old woman diagnosed with intracerebral haemorrhage (ICH); the other was a 28-year-old woman who had undergone curettage due to a failed abortion and died from sepsis because of perforations in the uterus and intestine.

The rate of maternal death in near miss cases was 0.40% with a case : fatality ratio of 250 : 1.

As shown in Table 3, the most prevalent causes of near miss were severe preeclampsia (27.3%), ectopic pregnancy (18.4%), and abruptio placentae (16.2%). In all, 15.2% had at least one systemic disease, and 43 women in the near miss group were hospitalised in the ICU (Table 3). The majority of the near miss cases were observed in the haemorrhagic disorders group (Table 3). Logistic regression analysis showed that four variables had significant relationship with near miss occurrence (Table 4). Of the 377 near miss cases (75.2%), the major problem was discerned at or during the first six hours of admission.

## 4. Discussion

In the present study, the near miss ratio was 25.2 per 1000 live births. In the private setting, this rate was 7.5 per 1000, and, in the tertiary care hospital, it was approximately 105 per 1000. The main advantages of this study were its prospective nature and its use of standard criteria for determining near miss cases. Because more than 97% of deliveries in Iran occur in hospitals [16], the present report may be considered as a population-based study. Because of the differences among patients and health care delivery systems, generalizing the results of this study on a countrywide basis should be done with caution.

The literature shows that maternal near miss ratios vary greatly depending on the population studied, how near miss is defined, and how the study is conducted (prospective versus retrospective) [11, 19]. Near miss ratios have been reported as 44.3 per 1000 in Brazil [9], 33 per 1000 in India [20], 3.83 per 1000 in Scotland [21], and 34 per 1000 in a WHO survey [10]. A recent systematic review using a unique definition for near miss showed SAMM rates in high-income countries to be significantly lower compared with those of low- and middle-income countries [11].

In this study, like some other studies [19, 20], the rate of near miss was significantly higher in the tertiary care setting (Table 1). The reason might be that, because of the limited facilities at private hospitals, women with complicated pregnancies are not usually referred to these centres.

In the present study, we found a higher rate of near miss and consequently a lower fatality rate (0.4%). These findings may be derived from our broader definition for near miss events, which combined disease-specific criteria with management-based criteria [8]. In Netherland, with a near miss ratio of 7.1 per 1000, the rate was 1.9% [22]. In Brazil, with a near miss ratio of 42 per 1000, the rate was 1.6% [19]. It should be mentioned that the maternal death rate shows a decreasing trend in Iran [12].

In our study, haemorrhagic disorders (46.1%) and hypertensive disorders (31.9%) were the most common causes of near miss (Table 3). These rates are similar to those reported from Scotland [21] and Indonesia [23]. In India, the two most common causes of near miss have been preeclampsia and haemorrhage [20]. In a study on 64 cases of maternal death in Kerman, Iran, the same two factors were the most prevalent causes of maternal mortality [15]. In our study, 43 cases required intensive care; that is, for every 1000 live births, 2.2 mothers are hospitalized in an ICU, which is nearly similar to the ICU admission rate in Netherland [22].

According to our logistic regression model, four variables had a relationship with near miss (Table 4). In older women with university degrees, the near miss ratio was higher. In fact, most studies demonstrate a proportional relationship between higher near miss ratio and advancing age (particularly over 35 years) [22, 23]. In the WHO's global survey, the near miss ratio was significantly associated with higher educational levels. This finding has been attributed to the tendency among women with higher educational levels to undergo caesarean section, which increases the probability of near miss events [22]. The present study, as in previous studies, shows that being a primipara increases the probability of near miss ratio by 1.2%–1.4% [22, 23].

Although pregnancy complications are to a great extent unpredictable and unpreventable, early awareness of near miss cases can prevent the progress of disease and maternal death [19]. In this study, the feasibility of using WHO-recommended near miss criteria was recognized. However, because of inadequate health care services, it is necessary that the auditing of near miss cases be considered as important as the implementation of near miss surveillance systems [15, 23].

The present study showed that using data related to near miss cases can provide more comprehensive information when reviewing maternal death cases; therefore, establishing near miss surveillance systems in Iranian hospitals is highly recommended.

## Figures and Tables

**Table 1:** Maternal near miss ratio according to hospital type.

Hospital type	Number of near miss cases	Number of live births	Ratio (CI 95%)
Referral	345	3293	104.8 (94.8–115.7)
Public	128	2877	9.9 (8.3–11.8)
Private	28	3738	7.5 (5.2–10.8)

Total	501	9908	25.2 (23.1–27.5)

**Table 2:** Baseline and clinical characteristics in near miss and control groups.

Variable	Total	Group		*P* value
		Near miss	Control	
		(*n* = 501)	(*n* = 498)	
Age group^*^ (yrs)				
<18	22	4 (18.2)	18 (81.8)	<0.001
18–35	978	432 (49.1)	447 (50.9)	
>35	98	65 (66.3)	33 (33.7)	
Education^*^				
≤Primary	251	127 (50.6)	124 (49.4)	<0.001
Secondary	576	260 (45.1)	316 (54.9)	
College	172	114 (66.3)	58 (33.7)	
Residence				
Urban	789	402 (51.0)	387 (49.0)	0.327
Rural	210	99 (47.1)	111 (52.9)	
Type of delivery^*^				
Normal vaginal delivery	501	134 (26.7)	367 (73.3)	<0.001
First cesarean delivery	254	189 (74.4)	65 (25.6)	
Repeat cesarean delivery	142	84 (59.2)	58 (40.8)	
Vaginal birth after cesarean delivery	1	1 (100)	0 (0)	
Type of abortion^*^				
Medical	54	51 (94.4)	3 (5.6)	0.545
Surgical	45	40 (88.9)	5 (11.1)	
Criminal	2	2 (100)	0 (0)	
Gravidity (mean ± SE)	—	2.3 (0.06)	2.1 (0.06)	0.012
Parity (mean ± SE)	—	1.1 (0.06)	1.0 (0.06)	0.048
Abortion (mean ± SE)	—	1.2 (0.08)	1.2 (0.07)	0.460
Living (mean ± SE)	—	2.3 (0.3)	1.7 (0.09)	0.058
Birth interval (mean ± SE)	—	5.3 (0.2)	4.7 (0.2)	0.055
Gestational age (mean ± SE)	—	27.7 (0.6)	34.8 (0.4)	<0.001
Number of prenatal care (mean ± SE)	—	6.4 (0.2)	6.9 (0.2)	0.028

^*^Numbers in parentheses are percents.

**Table 3:** Frequency of near miss criteria in 501 cases of severe maternal morbidity.

Type of near miss	Frequency (%)
Hemorrhagic disorders	
Abruptio placentae	81 (16.2)
Accreta/increta/percreta placenta	12 (2.4)
Ectopic pregnancy	92 (18.4)
Postpartum haemorrhage	50 (10)
Ruptured uterus	3 (0.6)
At least one type	231 (46.1)
Hypertensive disorders	
Severe preeclampsia	137 (27.3)
Eclampsia	11 (2.2)
Severe hypertension (>170/110)	35 (7.0)
Hypertensive encephalopathy	0 (0)
HELLP syndrome	10 (2.0)
At least one type	160 (31.9)
Other systemic disorders	
Endometritis	12 (2.4)
Pulmonary edema	0 (0)
Respiratory failure	7 (1.4)
Seizures	12 (2.4)
Sepsis	4 (0.8)
Shock	5 (1.0)
Thrombocytopenia < 100000	39 (7.8)
Thyroid crisis	0 (0)
At least one type	76 (15.2)
Severe management indicators	
Blood transfusion ≥ 5 units	17 (3.4)
Central venous access	2 (0.4)
Hysterectomy	10 (2.0)
ICU admission	43 (8.6)
Prolonged hospital stay (>7 postpartum days)	28 (5.6)
Nonanesthetic intubation	5 (1.0)
Return to operating room	7 (1.4)
Surgical intervention (other than cesarean section & hysterectomy)	10 (2.0)
Dialysis for acute renal failure	1 (0.2)
Cardiopulmonary resuscitation (CPR)	2 (0.4)
Others (please specify)	12 (2.4)
At least one type	91 (18.2)

**Table 4:** Baseline characteristics associated with near miss cases.

Baseline variables	Adjusted odds ratio	CI 95%	*P* value
Age	1.08	1.05–1.11	<0.001
Education			
College	1.97	1.35–2.88	<0.001
Others	1	—	—
Primiparous			
Yes	1.41	1.03–1.95	0.033
No	1	—	—
Number of prenatal care	0.94	0.91–0.98	0.003
